# Dynamics of Borrelial Antibodies in European Lyme Borreliosis Patients with Erythema Migrans

**DOI:** 10.3390/medsci14050563

**Published:** 2026-09-12

**Authors:** Petra Bogovič, Martina Jaklič, Katarina Ogrinc, Vera Maraspin, Tereza Rojko, Mirijam Nahtigal-Klevišar, Eva Ružić-Sabljić, Gary P. Wormser, Franc Strle

**Affiliations:** 1 Department of Infectious Diseases, University Medical Centre Ljubljana, 1525 Ljubljana, Slovenia; petra.bogovic@kclj.si (P.B.); katarina.ogrinc@kclj.si (K.O.); vera.maraspin@kclj.si (V.M.); tereza.rojko@kclj.si (T.R.); mirijam.nahtigal@kclj.si (M.N.-K.); 2 Centre for Clinical Research, University Medical Centre Ljubljana, 1000 Ljubljana, Slovenia; martina.jaklic@kclj.si; 3 Institute of Microbiology and Immunology, Medical Faculty, University of Ljubljana, 1000 Ljubljana, Slovenia; eva.ruzic-sabljic@mf.uni-lj.si; 4 Division of Infectious Diseases, Department of Medicine, New York Medical College, Valhalla, NY 10595, USA; gwormser@nymc.edu

**Keywords:** Lyme borreliosis, erythema migrans, *Borrelia burgdorferi* sensu lato, humoral immune response, IgM antibodies, IgG antibodies, Lyme serology

## Abstract

**Background/Objectives:** The diagnosis of typical erythema migrans (EM) is clinical, and serological testing for *Borrelia*-specific antibodies is not required. However, characterization of the immune response in patients with a reliable clinical diagnosis may improve the interpretation of serological findings in early Lyme borreliosis (LB) without characteristic skin lesions. **Methods:** Serum IgM and IgG antibodies to *Borrelia burgdorferi* sensu lato were evaluated in 1356 Slovenian adults with EM at baseline and at a follow-up visit approximately two months after treatment initiation, using a chemiluminescence immunoassay based on recombinant borrelial antigens. **Results:** At baseline, 41.5% of patients were IgM-seropositive, 55.5% IgG-seropositive, and 70.1% positive for either IgM or IgG. Patients with multiple EM were significantly more often seropositive than those with solitary EM. In previously untreated patients, baseline IgG seropositivity showed a clearer positive association with longer lesion duration and larger lesion diameter, whereas the corresponding IgM patterns were less consistent. Multiple EM remained strongly associated with both IgM and IgG seropositivity in adjusted analyses. Seroconversion at follow-up was uncommon, occurring in 8.0% of initially IgM-negative and 8.2% of initially IgG-negative patients. **Conclusions:** The low frequency of newly detectable antibody responses indicates that repeat qualitative serologic testing approximately two months after treatment initiation provides limited additional diagnostic information in European adults with EM.

## 1. Introduction

Lyme borreliosis (LB) is the most common tick-borne disease in the Northern Hemisphere and is caused by several species within the *Borrelia burgdorferi* sensu lato complex (Lyme borreliae). The distribution of causative species differs geographically; in Europe, *Borrelia afzelii* and *Borrelia garinii* predominate, whereas *B. burgdorferi* sensu stricto is the major cause of LB in North America. These biologic differences contribute to differences in the clinical presentation of LB between Europe and North America [1,2].

Erythema migrans (EM) is the most common manifestation of LB and is sufficiently characteristic to permit a clinical diagnosis without laboratory confirmation [3,4,5,6]. Patients with clinically well-defined EM, nevertheless, provide a useful model for characterizing the humoral immune response in early LB.

Contemporary data on serologic responses in patients with EM obtained using modern recombinant-antigen assays remain relatively limited, particularly in Europe [7,8,9,10,11,12]. The diagnostic sensitivity of serologic testing depends strongly on symptom duration and on the clinical manifestation of LB [1,2,3,5,7,11,13,14].

Serum antibodies to Lyme borreliae develop with a time delay; consequently, serologic testing may be negative during the first days to weeks after disease onset. This limitation is particularly relevant in patients with EM [5,11,14]. Current guidelines recommend repeat serologic testing in selected patients with possible but atypical EM and initially negative serology, although recommendations differ somewhat between geographic regions [3,5,6,15].

Among untreated North American patients with solitary EM, seropositivity is low early in the illness and increases substantially with longer lesion duration. Seropositivity is also higher in patients with multiple EM lesions than in those with a solitary lesion [5,14,15].

Earlier North American studies demonstrated substantial evolution of the borrelial antibody response after treatment in patients with EM [12,16,17], whereas more recent studies have reported considerably lower seroconversion rates (≤4%) [10,17,18,19]. The reasons for these differences remain uncertain and may include differences in study populations, timing of presentation and treatment, diagnostic criteria, definitions of seroconversion, and serologic methods.

Comparable European data are limited [9,11,12,18,19,20]. European studies have shown substantial variation in seropositivity at presentation and in post-treatment antibody kinetics, with many patients remaining seronegative after treatment [1,7,13]. In routine practice at our Lyme borreliosis outpatient clinic, approximately one-half of patients with EM are IgG-seronegative at presentation, and subsequent seroconversion appears uncommon but has not previously been systematically evaluated.

The aim of this study was to characterize the dynamics of serum borrelial antibodies in European patients with EM using chemiluminescence serologic assays. Specifically, we sought to compare IgM and IgG seropositivity in patients with solitary and multiple EM at presentation, determine the frequency of seroconversion approximately two months after initiation of antibiotic treatment, and identify clinical factors associated with borrelial seropositivity at the two study timepoints.

## 2. Materials and Methods

We conducted an observational cohort study of adult patients with erythema migrans (EM), with clinical and serologic assessments at presentation and at a follow-up visit approximately two months later. All patients diagnosed with EM at the LB Outpatient Clinic in Slovenia between 2016 and 2020 who underwent serologic testing for antibodies to *B. burgdorferi* sensu lato at both visits were eligible for inclusion. Clinical and epidemiological data were collected prospectively using a structured questionnaire. Only adults (≥18 years) who were diagnosed and managed by the Slovenian clinicians who are co-authors of this report were included.

### 2.1. Definition of EM

EM was defined as an expanding erythematous skin lesion, with or without central clearing, that developed days to a few weeks after a tick bite or exposure to ticks in an LB-endemic area and had a maximum diameter of ≥5 cm. If <5 cm, diagnosis required a history of a tick bite at that exact skin site, a delay in appearance of the skin lesion for at least two days after the tick bite, and documented expansion of the skin lesion. Multiple EM was defined as the presence of ≥two EM skin lesions, at least one of which fulfilled the size criterion (≥5 cm) for a solitary EM. In patients with multiple EM skin lesions, the size of the primary EM skin lesion was evaluated. The primary EM was defined as the lesion at the site of the tick bite; if no tick bite was recalled, it was defined as the lesion with the longest duration; if duration was the same or uncertain, it was defined as the lesion with the largest diameter.

The date of onset of EM was defined as the day on which the lesion was first noticed by the patient. Duration of EM at time of presentation was calculated as the number of days from lesion onset to the initial visit to the LB Outpatient Clinic. Because EM lesions generally expand after onset, lesion diameter at presentation was examined as a complementary indirect marker related to time elapsed since lesion onset; it was not considered equivalent to reported EM duration because lesion growth rates vary among patients.

### 2.2. Antibiotic Treatment

All patients were treated with antibiotics. The most commonly prescribed antibiotics were doxycycline, followed by amoxicillin (Table 1); treatment with either doxycycline or amoxicillin lasted 14 days, although some patients (<10%) received doxycycline for only 10 days; in contrast, azithromycin was prescribed for 5 days.

### 2.3. Serologic Testing

Serum IgM and IgG antibodies against *B. burgdorferi* sensu lato were measured using indirect chemiluminescence immunoassays (LIAISON^®^, DiaSorin, Saluggia, Italy). Recombinant OspC and VlsE antigens were used for IgM detection, and recombinant VlsE for IgG detection. Numerical assay output was expressed in assay-specific arbitrary units (AU/mL), which should not be interpreted as absolute antibody concentrations. Results were classified as positive, borderline, or negative according to the manufacturer’s cut-offs (IgM: >21, 18–21, <18 AU/mL; IgG: >15, 10–15, <10 AU/mL).

For binary analyses, results were analytically classified as positive versus non-positive; the non-positive category comprised borderline and negative laboratory results. This analytical dichotomization did not reclassify borderline results as laboratory-negative. Strict seroconversion was defined as a transition from a negative result at baseline to a positive result at follow-up, and strict seroreversion as a transition from a positive result at baseline to a negative result at follow-up. Testing was performed at the initial visit and repeated approximately two months later (median 59 days, IQR 56–61 days after the first visit).

For complementary analyses of newly detectable seropositivity, transitions from the broader non-positive category (negative or borderline) to positive were also summarized separately from strict seroconversion.

### 2.4. Borrelia Cultivation and Typing

To test for the presence of *Borrelia* in skin, a 3 mm punch biopsy was obtained from the expanding edge of the EM skin lesion after local anesthesia. The skin biopsy specimen was immediately inoculated into a 10 mL tube with a modified Kelly–Pettenkofer (MKP) medium [9].

To test for the presence of *Borrelia* in blood, 9 mL of whole blood was obtained by venipuncture and placed into tubes containing sodium citrate. Blood was gently centrifuged (800 rpm), and 1 mL of plasma was inoculated into 7 mL modified MKP medium and incubated at 33 °C [9].

Cultures were examined weekly by dark-field microscopy for the presence of spirochetes for up to 9 weeks (skin) or 12 weeks (blood). The species of cultured isolates was determined using pulsed-field gel electrophoresis following *MluI* restriction of genomic DNA (*MluI*-large restriction fragment pattern, LRFP) or by PCR-based *MseI*-restriction fragment length polymorphism (*MseI*-RFLP) of the 5S–23S intergenic region [9].

### 2.5. Statistical Analysis

Continuous variables are presented as medians with interquartile ranges (IQRs), and categorical variables as frequencies and percentages. Comparisons between patients with solitary and multiple EM used the Mann–Whitney U test for continuous variables, Pearson’s chi-square test for categorical variables when expected cell counts were adequate, Fisher’s exact test for sparse 2 × 2 tables, and the Fisher–Freeman–Halton exact test for sparse larger contingency tables. For omnibus contingency-table analyses, the null hypothesis was that the distribution of categories was independent of solitary versus multiple EM. Changes in binary seropositivity between baseline and follow-up were assessed using McNemar’s test for paired data. All tests were two-sided.

Associations between demographic and clinical factors and IgM or IgG seropositivity were evaluated using multivariable logistic regression. Reported odds ratios (ORs) are mutually adjusted estimates conditional on all other covariates in the same model, with 95% confidence intervals (CIs). Two-sided coefficient-level Wald *p*-values were obtained from the fitted models; no adjustment for multiple comparisons across coefficients was applied. Age was scaled per 10-year increase. Multicollinearity was assessed using VIF/GVIF. Functional form for continuous predictors was assessed by comparing linear specifications with four-degree-of-freedom natural cubic-spline specifications; model discrimination and calibration were assessed using the area under the receiver-operating-characteristic curve (AUC), Brier score, and calibration tests.

As a post hoc longitudinal analysis, baseline and follow-up observations were combined in separate logistic generalized estimating-equation (GEE) models for IgM and IgG. Patient identifier defined the repeated-measures cluster; an exchangeable working correlation structure and robust sandwich standard errors were used. The models included timepoint, eight covariates available at both measurements, and the corresponding timepoint-by-covariate interactions; continuous covariates were mean-centered. In a complementary baseline analysis, numerical AU/mL assay values were log10-transformed and analyzed with multivariable linear regression using the same covariates and complete-case sample as the baseline logistic models; left-censored Tobit regression was used as a sensitivity analysis because some values were recorded at the lower assay limit. All analyses were performed in R version 4.3.2 (R Foundation for Statistical Computing, Vienna, Austria). Forest plots and descriptive smooths were generated using ggplot2.

### 2.6. Ethics

The study was conducted in accordance with the Declaration of Helsinki and was approved by the Medical Ethics Committee of the Republic of Slovenia (No. 0120-458/2025-2711-3; 24 September 2025). The Ethics Committee waived the need for written informed consent. However, all patients in whom a skin biopsy was performed gave written informed consent for the procedure as a part of the routine clinical practice (good clinical practice).

## 3. Results

Of the 1850 patients diagnosed with EM at our LB Outpatient Clinic during the 5-year study period, 1356 (73.3%) met the inclusion criteria. The others either did not undergo serologic testing for antibodies to *B. burgdorferi* sensu lato at the initial visit and again approximately two months later, or were not examined by one of the clinicians from the Clinic who are co-authors of this report, or were <18 years old. The cohort that was studied included 815 women (60.1%) and 541 men (39.9%), with a median age of 55 (IQR 42–63) years. A solitary EM skin lesion was present in 1111 patients (81.9%), whereas 245 (18.1%) had multiple EM skin lesions. The main demographic, clinical, and epidemiologic characteristics of the study population are presented in Table 1.

*Borrelia* was isolated from skin in 267 of 897 (29.8%) cultured samples and from blood in 22 of 1226 (1.8%) cultured samples. Among isolates for which species identification was available, more than 85% of both skin and blood isolates were identified as *B. afzelii*; *B. afzelii* also predominated among isolates from both patients with solitary EM and those with multiple EM. Because the numbers of isolates belonging to other *Borrelia* species were small, species-specific analyses of serologic responses were not performed.

### 3.1. Serologic Findings at the Initial Visit

Among the 1356 patients, at the baseline visit 563 (41.5%) were IgM-seropositive, 77 (5.7%) had borderline IgM test results, and 716 (52.8%) were IgM-seronegative. The corresponding IgG results were 752 (55.5%) positive, 130 (9.6%) borderline, and 474 (35.0%) IgG-seronegative. For binary analyses, borderline and negative results were combined in the non-positive category. At baseline, 951 of 1356 patients (70.1%) were positive for IgM and/or IgG.

At baseline, the most common joint serologic pattern was non-positive IgM/non-positive IgG (29.9%), whereas positive IgM/non-positive IgG was the least common (14.7%). The distribution differed between solitary and multiple EM (Supplementary Table S1). In patients with solitary EM, the most frequent pattern was non-positive IgM/non-positive IgG (33.4%); in multiple EM, the most frequent pattern was positive IgM/positive IgG (53.5%).

Compared to patients with solitary EM, those with multiple EM were significantly more often IgM-positive (67.8% vs. 35.7%; chi-square = 84.77, df = 1, *p* < 0.001), IgG-positive (71.8% vs. 51.8%; chi-square = 32.48, df = 1, *p* < 0.001), and positive for IgM and/or IgG (86.1% vs. 66.6%; chi-square = 36.50, df = 1, *p* < 0.001) at baseline (Table 2).

#### 3.1.1. Previous Antibiotic Treatment

At the first clinic visit, 116 patients (8.6%) were already receiving antibiotics active against borreliae for a median of 5 days (IQR 2–5, range: 1–16) (Supplementary Table S2). The distribution of the antibiotics received before the first serologic assessment is shown in Supplementary Table S2. Baseline IgM and IgG seropositivity according to prior antibiotic exposure are presented separately in Supplementary Table S3; prior treatment was associated with a lower proportion of IgG-seropositive patients, whereas the difference in IgM seropositivity was not statistically significant.

#### 3.1.2. Duration and Diameter of Erythema Migrans at Presentation

The descriptive analyses according to EM duration and lesion diameter were restricted to the 1190 patients who had not received antibiotics before the initial serologic assessment; in contrast, the general baseline summaries and multivariable models included both previously treated and untreated patients. Among untreated patients presenting at different intervals after EM onset, IgM seropositivity was more frequent in groups presenting during the second week than in groups presenting earlier, whereas the pattern was less consistent at longer durations. These are between-patient, cross-sectional differences; each patient contributed one baseline observation to this analysis.

**Figure 1:**
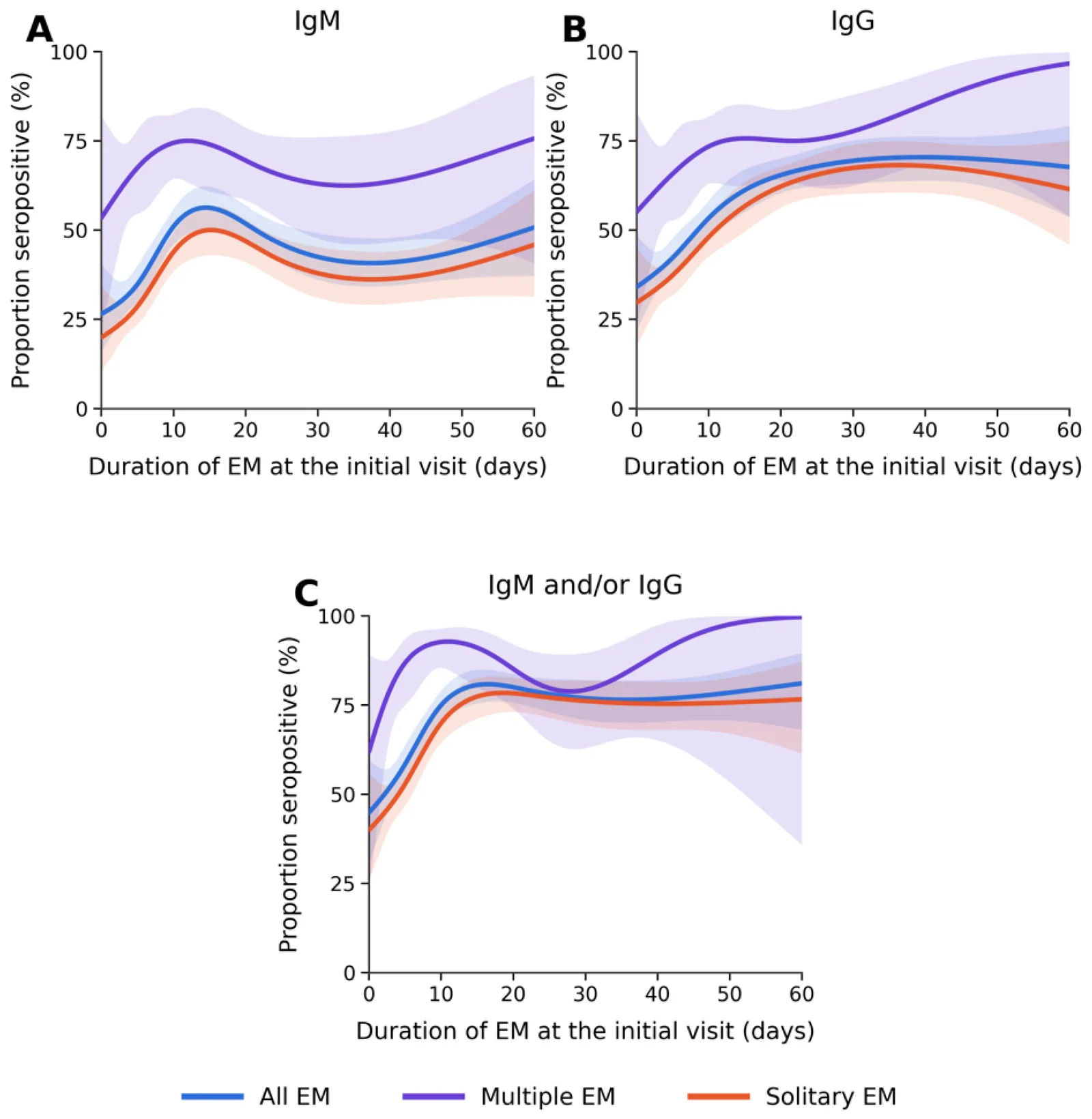
Cross-sectional proportion of untreated patients who tested positive for borrelial antibodies at the initial visit according to reported duration of erythema migrans at presentation. Each patient contributed one baseline observation; the figure should not be interpreted as a within-patient longitudinal trajectory. (**A**) IgM antibodies; (**B**) IgG antibodies; (**C**) IgM and/or IgG antibodies.

Across untreated patients presenting at different intervals after EM onset, IgG seropositivity was generally more frequent with longer reported lesion duration, particularly in multiple EM. At the longest observed durations, estimates were based on relatively few patients and should be interpreted cautiously. These cross-sectional patterns do not represent within-patient evolution of the antibody response (Figure 1).

**Figure 2:**
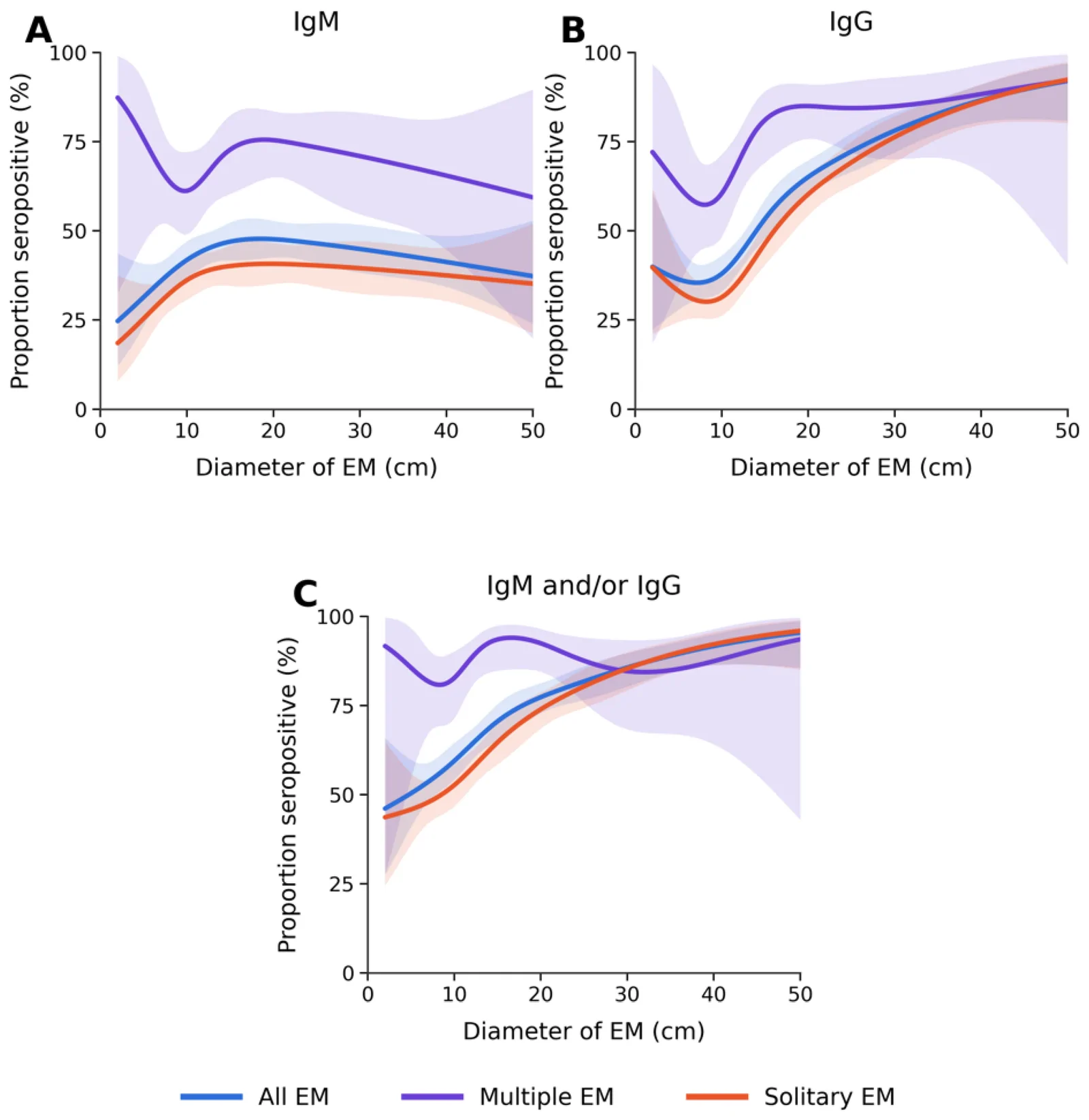
The proportion of patients with positive borrelial antibodies in serum according to the largest diameter of erythema migrans. (**A**) IgM antibodies; (**B**) IgG antibodies; (**C**) IgM and/or IgG antibodies.

In the same untreated subgroup at presentation, IgG seropositivity showed a clearer positive association with larger lesion diameter, whereas the corresponding IgM pattern was less consistent (Figure 2). Lesion diameter was moderately correlated with reported EM duration at presentation (Spearman rho = 0.42, *p* < 0.001). Both IgM and IgG seropositivity were significantly more frequent in patients with multiple than solitary EM (IgM: chi-square = 84.77, df = 1, *p* < 0.001; IgG: chi-square = 32.48, df = 1, *p* < 0.001; Table 2).

#### 3.1.3. Logistic Regression Analysis

**Figure 3:**
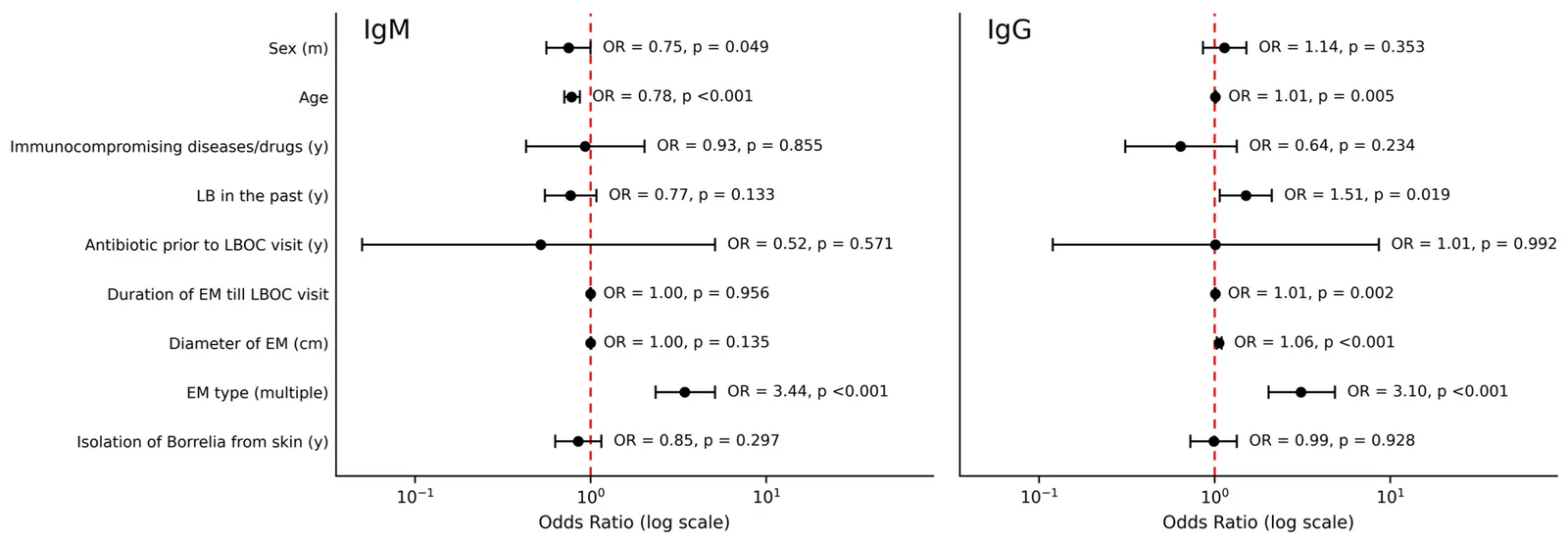
Variables associated with borrelial seropositivity at presentation.

The baseline multivariable analyses included patients evaluated at the initial visit irrespective of whether antibiotic treatment had been started before presentation; prior antibiotic treatment was included as a covariate. Multiple EM remained strongly associated with IgM seropositivity, and IgM seropositivity was also more common in women and younger patients. Diagnostic analyses showed no problematic multicollinearity (all VIF values ≤ 1.291) but indicated nonlinearity for some continuous predictors; therefore, per-unit linear ORs should not be interpreted as constant effects across the full observed range (Figure 3; Supplementary Table S4).

For IgG, seropositivity was associated with multiple EM, older age, prior LB, longer reported EM duration, and larger lesion diameter, but not sex. Natural cubic-spline diagnostics indicated evidence of nonlinearity for EM duration and lesion diameter, supporting cautious interpretation of simple per-unit linear (Figure 3; Supplementary Table S5).

### 3.2. Serologic Findings Approximately Two Months After the Initial Examination

At follow-up approximately two months after the initial visit (median 59 days, IQR 56–61 days), IgM and IgG seropositivity rates remained significantly higher in patients with multiple EM than in those with solitary EM (Supplementary Table S6).

**Figure 4:**
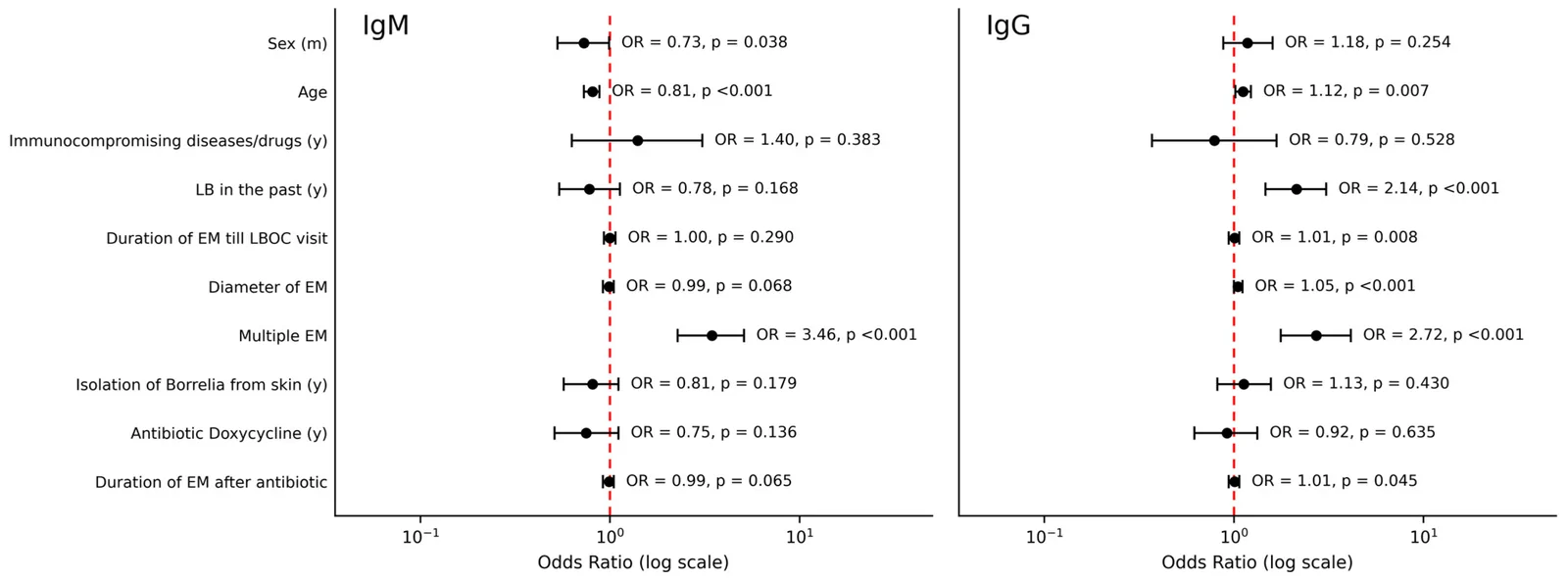
Variables associated with borrelial antibody seropositivity at approximately 2 months after the initial examination (i.e., also approximately 2 months after the initiation of antibiotic treatment).

At follow-up, IgM seropositivity remained associated with female sex, younger age, and multiple EM, whereas IgG seropositivity was associated with older age, previous LB, baseline EM duration, lesion diameter, multiple EM, and longer persistence of EM after antibiotic initiation (Figure 4; Supplementary Tables S7 and S8). In the post hoc GEE analysis of repeated measurements, the estimated within-patient working correlation was 0.695 for IgM and 0.751 for IgG. The global timepoint-by-covariate interaction test was not statistically significant for IgM (chi-square = 14.84, df = 8, *p* = 0.062) but was significant for IgG (chi-square = 17.97, df = 8, *p* = 0.022). Multiple EM remained a strong predictor of both measurements without a significant interaction with timepoint.

### 3.3. Comparison of Serologic Test Results at the Initial Visit and at the Visit Approximately 2 Months Later

The follow-up serologic testing was performed at a median of 59 days (IQR 56–61 days), and ranged 39–70 days after the initial visit.

#### 3.3.1. Seropositivity

In the overall cohort, IgM and IgG seropositivity were slightly but statistically significantly lower at follow-up than at baseline (IgM: McNemar chi-square = 10.52, df = 1, *p* = 0.001; IgG: chi-square = 4.90, df = 1, *p* = 0.027; Supplementary Table S9). In a sensitivity analysis restricted to patients with strictly positive or strictly negative results at both visits, excluding borderline results, neither change was statistically significant (IgM: chi-square = 2.21, df = 1, *p* = 0.138; IgG: chi-square = 0.30, df = 1, *p* = 0.583).

#### 3.3.2. Seroconversion and Seroreversion

Among 793 patients who were non-positive for IgM at baseline (negative or borderline), 70 (8.8%) were IgM-positive at follow-up. Among 604 patients who were non-positive for IgG at baseline, 66 (10.9%) were IgG-positive at follow-up. These transitions are reported as newly detectable seropositivity and are distinct from strict seroconversion.

Strict seroconversion from a negative baseline result to a positive follow-up result occurred in 57/716 (8.0%) for IgM and 39/474 (8.2%) for IgG. Only nine/1356 patients (0.7%) developed both IgM and IgG positivity at follow-up.

Among 199 patients with an initial IgM-positive/IgG-negative profile, which is typically considered compatible with early infection, 37 (18.6%) subsequently showed IgG seroconversion. Among 388 patients with an initial IgM-negative/IgG-positive profile, five (1.3%) subsequently showed IgM seroconversion (*p* < 0.001).

Seroreversion (from initially positive to borderline or negative) occurred in 114 of 563 initially IgM-seropositive patients (20.2%) and in 94 of 752 initially IgG-seropositive patients (12.5%) at the 2-month assessment (all had received antibiotic treatment).

Seroreversion (from initially positive to negative only and excluding to borderline) occurred in 74 of 563 initially IgM-seropositive patients (13.1%) and in 44 of 752 initially IgG-seropositive patients (5.9%) at the 2-month assessment.

## 4. Discussion

The present study characterizes serum IgM and IgG responses in a large cohort of European patients with clinically defined EM using a contemporary recombinant-antigen chemiluminescence assay. The large number of patients with both solitary and multiple EM enabled the detailed assessment of serologic responses according to disease presentation and selected clinical characteristics.

The clearest and most consistent distinction was observed between solitary EM, which usually reflects localized infection, and multiple EM, which is generally considered a manifestation of early disseminated disease. Patients with multiple EM were substantially more often IgM- and IgG-seropositive both at presentation and approximately two months later. These associations remained evident after adjustment for lesion duration and other clinical characteristics, supporting a strong association between early dissemination and the detectable humoral response. Differences in seropositivity rates between solitary and multiple EM have also been reported previously [5,14,15,22,23].

The overall clinical profile of our Slovenian cohort was largely consistent with previous European studies [22,24,25,26,27,28,29]. Two observations deserve comment. First, only 39% of patients recalled a tick bite at the site of the EM skin lesion, which is lower than in several previous European reports [24,26,27,28]. Second, the proportion of patients with multiple EM was relatively high (18%); however, higher proportions have been reported in selected European referral cohorts [22,23,29], probably reflecting the referral patterns to our LB Center in Slovenia. This is relevant because studies that combine solitary and multiple EM may be strongly influenced by the predominance of solitary EM in Europe.

The relatively large number of patients with multiple EM is an important strength of the present study because it allowed a robust direct comparison with solitary EM within the same clinical setting and time period. In agreement with previous observations, patients with multiple EM less often recalled a tick bite and less often reported local symptoms at the EM skin sites, whereas constitutional symptoms and positive blood cultures were more frequent [22,23]. The higher rate of positive blood cultures in multiple EM reported in prior studies [30] and in the current study supports the interpretation that this presentation reflects current or recent hematogenous dissemination. Nevertheless, the overall frequency of a positive blood culture remained substantially lower than that reported in North American studies [30,31], in line with known biologic differences between European and North American Lyme borreliae [32,33]. However, the culture methodology used is also different in the USA [30]. Skin culture and serology assess different biological phenomena. A positive skin culture is highly specific but culture sensitivity is limited; in this cohort *Borrelia* was isolated from 267/897 (29.8%) cultured skin samples. Failure to recover *Borrelia* therefore does not imply the absence of infection. Conversely, seropositivity reflects the host humoral response and is influenced by disease duration, prior exposure, dissemination, and host factors. Accordingly, the absence of an independent adjusted association between skin-culture positivity and seropositivity does not imply that the two measurements are uncorrelated.

Cross-sectional analysis of untreated patients at study entry showed that the proportion of seropositive differed according to EM duration at presentation. IgG seropositivity showed a clearer positive association with longer reported EM duration, particularly in multiple EM, whereas the corresponding IgM pattern was less consistent. Because different patients contributed observations at different durations, these findings describe between-patient differences and should not be interpreted as the within-patient evolution of the antibody response. The relatively sparse observations at the longest durations further limit interpretation of the right-hand tail of the curves.

A similar distinction was observed for lesion diameter, which was examined as a complementary indirect marker related to time since lesion onset rather than as a direct proxy for disease duration. Larger lesions were associated more clearly with IgG seropositivity than with IgM seropositivity. Lesion diameter was only moderately correlated with reported EM duration, and lesion growth rates vary among patients and by the causative *Borrelia* species [2,26,29,34]; therefore, lesion size and reported duration should not be considered interchangeable.

A prior history of LB was associated with higher IgG seropositivity rates both at baseline and at 2 months. This finding is biologically plausible and consistent with having seropositivity antedating the current Lyme episode, as well as with the notion that previous exposure primes subsequent humoral responses. In contrast, the impact on the seropositivity of antibiotic treatment before the first visit was limited. Patients who had already started on antibiotic therapy tended to have lower seropositivity rates, but the difference was generally small and not confirmed in multivariate analyses. Thus, although early antibiotic therapy may attenuate antibody development [16,18,35], its effect in this cohort appeared modest.

Multivariate analyses further refined the interpretation of serologic responses. Baseline IgM seropositivity was associated with multiple EM and was more common in women and in younger patients. This pattern is consistent with known sex- and age-related differences in humoral immune responses [36,37]. In contrast, IgG seropositivity was associated with older age, longer lesion duration, larger lesion diameter, prior LB, and multiple EM. Thus, the multivariable findings were consistent with the distinct IgM and IgG seroreactivity patterns described above.

At the two-month follow-up visit, overall IgM and IgG seropositivity rates were slightly but statistically significantly lower than at baseline in the primary binary analysis. The post hoc longitudinal GEE analysis did not support a simple conclusion that all covariate associations were unchanged over time: the global interaction test was significant for IgG but not IgM. Multiple EM, however, remained strongly associated with seropositivity at both measurements without a significant interaction with timepoint.

One of the clinically most relevant observations of this study is the low frequency of newly detectable antibody responses after treatment. Among patients who were non-positive at baseline, 8.8% became IgM-positive and 10.9% became IgG-positive at follow-up. Using the stricter laboratory definition of seroconversion as negative-to-positive, the proportions were 8.0% for IgM and 8.2% for IgG. Even among patients with an initial IgM-positive/IgG-negative profile, subsequent IgG seroconversion occurred in fewer than one in five. These low rates provide the principal evidence that repeat qualitative serology approximately two months after treatment initiation adds limited diagnostic information in this cohort.

The proportions of seroconversions observed in the present study are substantially lower than those reported in several North American prospective studies of EM, in which seroconversion was observed considerably more frequently [3,12,14,16,31]. A plausible explanation is that the European *Borrelia* species induce a less intense inflammatory response than *B. burgdorferi* sensu stricto in North America, consistent with known clinical differences between the two geographic locations [2,33], as well as with experimental and immunological data [32,33]. Differences in the timing of presentation likely also contribute. In USA studies reporting higher seroconversion rates, patients were typically initially evaluated earlier (around day 7 of illness), whereas in the present study patients presented later (approximately day 14) [12,16,31]. As a result, the immune response in our cohort was already more developed at baseline, leading to a higher initial seropositivity rate and less opportunity for subsequent seroconversion. Consistent with the limited frequency of new seropositivity, the overall paired analysis showed only small changes in seropositivity over follow-up. Notably, more recent USA studies using two-tier serologic testing have reported very low seroconversion rates (≤4%) in treated patients regarded as having EM [10,17], which is unexpected and thus far unexplained.

Seroreversion was more common than strict seroconversion and was somewhat more common for IgM than for IgG, consistent with the more transient nature of IgM responses. Overall, qualitative serology showed limited short-term dynamic change after treatment. Together with the low frequency of strict seroconversion, this supports the conclusion that repeat qualitative serology at approximately two months provides limited additional diagnostic information in European adults with EM. Although seroconversion provides laboratory evidence of the development of a detectable antibody response and, in an appropriate clinical context, may support evidence of recent *Borrelia* infection, such confirmation is generally not required in patients with typical EM, for whom the diagnosis is clinical. Importantly, post-treatment changes in serologic status should not be used to assess treatment efficacy: seroreversion is not required to document successful treatment, whereas persistent seropositivity or subsequent seroconversion does not, by itself, indicate treatment failure [35,38,39,40,41,42]. Serologic follow-up is therefore not recommended for monitoring the response to antibiotic treatment [38,39,40,41,42]. Nevertheless, the characterization of antibody dynamics in patients with clinically well-defined EM may be useful beyond EM itself. Because these patients provide a reliable model of early LB, the observed patterns may facilitate the interpretation of serologic findings in patients with other suspected early manifestations of LB without characteristic skin lesions, in whom serologic evidence may have a more important role in the diagnostic assessment.

The current study has several strengths. It included a large, well-characterized cohort of adult patients with prospectively collected clinical data, standardized diagnostic criteria, and uniform use of the same serologic assay over the five-year study period. One of the potential challenges in a study on EM is to ensure that the diagnosis of EM is reliable. Since in our study patients with suspected EM were examined by physicians with extensive clinical experience and in-depth knowledge of LB, and since the definition of EM was strictly followed in this study, these issues should not be a concern for this investigation.

The study also has limitations. Only one commercial serologic assay was used, and although contemporary recombinant-antigen assays share important principles, performance varies across platforms and testing algorithms [6,7,41,42,43,44,45,46]; therefore, the findings cannot be extrapolated to all serologic platforms. Most importantly, acute and convalescent serologic testing was not performed at the exact same time using identical lots of the test kits. In addition, the results apply primarily to adults in a European setting and may not be directly generalizable to children in Europe or to patients with EM in North America. They may also be less generalizable to markedly immunosuppressed patients, in whom antibody responses to EM can differ [47].

## 5. Conclusions

This large single-center study conducted in Slovenia provides detailed information on early borrelial antibody responses in European adults with EM. Multiple EM was the factor most consistently associated with both IgM and IgG seropositivity, whereas longer lesion duration, larger lesion size, older age, and previous LB were primarily associated with IgG seropositivity. Strict seroconversion during the approximately two-month follow-up period was uncommon. These findings indicate that repeat qualitative serologic testing approximately two months after treatment initiation provides limited additional diagnostic information in European adults with EM.

## Figures and Tables

**Table 1:** The main demographic, epidemiologic and clinical characteristics of the 1356 patients with erythema migrans included in this study.

Characteristic	All EM (*n* = 1356)	Solitary EM (*n* = 1111)	Multiple EM (*n* = 245)	Test Statistic (df)	*p*–Value ^a^
Age (years)	54 (42–63)	54 (42–64)	52 (42–62)	U = 144,743.5	0.119
Sex, female	815 (60.1%)	670 (60.3%)	145 (59.2%)	χ^2^ = 0.105 (1)	0.745
male	541 (39.9%)	441 (39.7%)	100 (40.8%)		
Underlying chronic diseases ^b^	667 (49.2%)	548 (49.3%)	119 (48.6%)	χ^2^ = 0.046 (1)	0.831
Immunocompromising diseases/drugs ^c^	67 (4.9%)	56 (5.0%)	11 (4.5%)	Fisher’s exact	0.871
LB in the past	283/1252 (22.6%)	244/1028 (23.7%)	39/224 (17.4%)	χ^2^ = 4.205 (1)	0.040
Pregnancy	33/815 (4.1%)	31/670 (4.6%)	2/145 (1.4%)	Fisher’s exact	0.100
Tick bite ^d^	533 (39.3%)	454 (40.9%)	79 (32.2%)	χ^2^ = 6.251 (1)	0.012
Antibiotic treatment prior to LBOC visit	116 (8.6%)	100 (9.0%)	16 (6.5%)	χ^2^ = 1.566 (1)	0.211
Duration of prior antibiotic treatment (days)	5 (2–8)	5 (2–9)	4 (1–8)	U = 928.0	0.305
Duration of EM until LBOC visit (days)	13 (6–30) (*n* = 1327)	13 (6–30) (*n* = 1086)	12 (6–23) (*n* = 241)	U = 135,496.0	0.389
Diameter of EM (cm) ^e^	15 (10–24)	15 (10–25)	14 (10–22)	U = 140,754.0	0.160
Central clearing	657/1327 (49.5%)	535/1091 (49.0%)	122/236 (51.7%)	χ^2^ = 0.548 (1)	0.459
Local symptoms at EM site	494/1348 (36.6%)	420/1106 (38.0%)	74/242 (30.6%)	χ^2^ = 4.678 (1)	0.031
Constitutional symptoms (NOIS)	438/1356 (32.3%)	339/1111 (30.5%)	99/245 (40.4%)	χ^2^ = 8.988 (1)	0.003
Antibiotic treatment				χ^2^ = 71.891 (4)	<0.001
amoxicillin	188 (13.9%)	187 (16.8%)	1 (0.4%)		
azithromycin	67 (4.9%)	65 (5.9%)	2 (0.8%)		
ceftriaxone	48 (3.5%)	36 (3.2%)	12 (4.9%)		
cefuroxime	41 (3.0%)	40 (3.6%)	1 (0.4%)		
doxycycline	1012 (74.6%)	783 (70.5%)	229 (93.5%)		
Duration of EM after initiation of antibiotic treatment (days)	14 (6–28) (*n* = 1132)	14 (6–28) (*n* = 922)	11 (5–21) (*n* = 210)	U = 104,796.0	0.061
Isolation of *Borrelia* from skin	267/897 (29.8%)	220 ^f^/735 (29.9%)	47 ^g^/162 (29.0%)	χ^2^ = 0.054 (1)	0.817
Isolation of *Borrelia* from blood	22/1226 (1.8%)	12 ^h^/1003 (1.2%)	10 ^i^/223 (4.5%)	Fisher’s exact	0.003

Data are *n* (%) unless otherwise stated; continuous variables are median (IQR). χ^2^ = Pearson’s chi–square statistic; U = Mann–Whitney U statistic. Values in parentheses are degrees of freedom; neither U nor Fisher’s exact test has degrees of freedom. *p*–values are two-sided. *n*, number of cases; EM, erythema migrans; LB, Lyme borreliosis; LBOC, Lyme borreliosis outpatient clinic; NOIS, new or increasing symptoms such as headache, myalgia, arthralgia, and fatigue; IQR, interquartile range. ^a^ Comparison of findings in patients with solitary and multiple EM skin lesions. Pearson’s chi-square test was used for categorical variables when expected cell counts were adequate, Fisher’s exact test for lower numbers 2 × 2 tables, and the Mann–Whitney U test for continuous variables. ^b^ Such as cardiovascular, pulmonary, renal, liver, and/or metabolic conditions. ^c^ Moderate to severe immunocompromising conditions as previously defined [21]. ^d^ At the site of, and preceding, the EM skin lesion. ^e^ Largest diameter of the EM skin lesion; for multiple EM, the diameter of the primary lesion. ^f^ Of 164 strains that had been typed, 149 (91%) were *B. afzelii*. ^g^ Of 42 strains that had been typed, 36 (86%) were *B. afzelii*. ^h^ Of 11 strains that had been typed, 10 (91%) were *B. afzelii*. ^i^ Of 9 strains that had been typed, 8 (89%) were *B. afzelii*.

**Table 2:** Serologic findings in patients with erythema migrans at the baseline visit.

Seropositivity	All EM (*n* = 1356)	Solitary EM (*n* = 1111)	Multiple EM (*n* = 245)	Test Statistic (df)	*p*–Value ^a^
IgM	563 (41.5)	397 (35.7)	166 (67.8)	χ^2^ = 84.770 (1)	<0.001
IgG	752 (55.5)	576 (51.8)	176 (71.8)	χ^2^ = 32.477 (1)	<0.001
IgM and/or IgG	951 (70.1)	740 (66.6)	211 (86.1)	χ^2^ = 36.499 (1)	<0.001

Data are *n* (%). Positive results were compared with not-positive results; borderline and negative results were combined in the not-positive category. χ^2^ = Pearson’s chi-square statistic; values in parentheses are degrees of freedom. *p*-values are two-sided. *n*, number of cases; EM, erythema migrans. ^a^ Comparison of findings in patients with solitary and multiple erythema migrans using Pearson’s chi-square test.

## Data Availability

The original contributions presented in this study are included in the article/Supplementary Materials. Further inquiries can be directed to the corresponding author.

## Supplementary Materials

The following supporting information can be downloaded at: https://www.mdpi.com/article/10.3390/medsci14050563/s1; Table S1: Patterns of IgM and IgG antibody responses in 1356 patients with erythema migrans at the initial examination: Comparison of findings in patients with solitary and multiple erythema migrans; Table S2: Receiving antibiotics to which *Borrelia* is in vitro susceptible prior to the first serological testing; Table S3: Serologic findings in patients with erythema migrans at the initial examination according to prior treatment with antibiotics (median 5 days; IQR 2–5 days, range 1–16 days); Table S4: Multivariable logistic regression analysis of factors associated with IgM seropositivity at the initial visit to the Slovenian Lyme Borreliosis Outpatient Clinic; Table S5: Multivariable logistic regression analysis of factors associated with IgG seropositivity at the initial visit to Lyme Borreliosis Outpatient Clinic; Table S6: Serologic findings in patients with erythema migrans approximately 2 months after the initial examination and the initiation of antibiotic treatment; Table S7: Multivariable logistic regression analysis of factors associated with IgM seropositivity 2 months later (i.e., 2 months after the onset of antibiotic treatment); Table S8: Multivariable logistic regression analysis of factors associated with IgG seropositivity 2 months later (i.e., 2 months after the onset of antibiotic treatment); Table S9: Paired comparison of seropositivity at baseline and at the follow-up visit approximately two months later.

## Abbreviations

The following abbreviations are used in this manuscript:

EM	erythema migrans
LB	Lyme borreliosis
OspC	Outer surface protein C
VlsE	variable major protein-like sequence, expressed
IQR	interquartile range
MKP	modified Kelly–Pettenkofer
LRFP	large restriction fragment pattern, LRFP
RFLP	restriction fragment length polymorphism
OR	odds ratio
CI	confidence interval
LBOC	Lyme borreliosis outpatient clinic
NOIS	new or increasing symptoms
