# Supplementary material for: Dynamics of Borrelial Antibodies in European Lyme Borreliosis Patients with Erythema Migrans
Source: Med Sci (Basel). 2026 Sep 12;14(5):563. doi: 10.3390/medsci14050563 (PMC13609033; doi:10.3390/medsci14050563)
Supplement: Supplementary file 1 [file medsci-14-00563-s001.zip › medsci-4502143-supplementary.pdf]

Supplementary Table S1. Patterns of IgM and IgG antibody responses in 1356 patients with erythema migrans at the initial examination: Comparison of findings in patients with solitary and multiple erythema migrans

**Combinations**	**All EM** (*n* = 1356)	**Solitary EM** (*n* = 1111)	**Multiple EM** (*n* = 245)	Test statistic (df); P-value
IgM pos, IgG pos	364 (26.8)	233 (21.0)	131 (53.5)	chi-square = 115.57 (df = 3); p < 0.001
IgM non-pos, IgG non-pos	405 (29.9)	371 (33.4)	34 (13.9)	
IgM pos, IgG non-pos	199 (14.7)	164 (14.8)	35 (14.3)	
IgM non-pos, IgG pos	388 (28.6)	343 (30.9)	45 (18.4)	

Data are shown as number (%).

pos, positive; non-pos, non-positive (borderline and negative results combined for this binary pattern analysis).

[a] Pearson's chi-square test of the complete 4 × 2 table comparing the distribution of the four joint IgM/IgG patterns between solitary and multiple EM.

Supplementary Table S2. Receiving antibiotics to which *Borrelia* is in vitro susceptible prior to the first serological testing

Antibiotic treatment prior to the first serological testing	All EM (*n* = 116)	Solitary EM (*n* = 100)	Multiple EM (*n* = 16)	Exact test; P-value
amoxicillin	27 (23.3%)	25 (25.0%)	2 (12.5%)	Fisher-Freeman-Halton exact; p = 0.398
azithromycin	75 (64.7%)	64 (64.0%)	11 (68.8%)	
ceftriaxone	1 (0.9%)	1 (1.0%)	0 (0.0%)	
flucloxacillin	2 (1.7%)	2 (2.0%)	0 (0.0%)	
phenoxymethylpenicillin	2 (1.7%)	2 (2.0%)	0 (0.0%)	
doxycycline	9 (7.8%)	6 (6.0%)	3 (18.8%)	

Data are shown as number (%)

n, number of cases; EM, erythema migrans.

[a] Fisher-Freeman-Halton exact test of the complete 6 x 2 table; no conventional chi-square statistic or degrees of freedom are reported for this exact test.

Supplementary Table S3. Serologic findings in patients with erythema migrans at the initial examination according to prior treatment with antibiotics (median 5 days; IQR 2–5 days, range 1 –16 days)

Prior treatment with antibiotics	**No** (*n* = 1240)	**Yes** (*n* = 116)	Test statistic (df); P-value
**Seropositivity**			
IgM	523 (42.2%)	40 (34.5%)	chi-square = 2.28 (df = 1); p = 0.131
IgG	699 (56.4%)	53 (45.7%)	chi-square = 4.48 (df = 1); p = 0.034

Data are shown as number (%).

n, number of patients.

[a] Pearson's chi-square test comparing positive versus non-positive results between patients who had and had not received antibiotics before serologic testing; borderline and negative results were combined in the non-positive category.

Supplementary Table S4. Multivariable logistic regression analysis of factors associated with IgM seropositivity at the initial visit to the Slovenian Lyme Borreliosis Outpatient Clinic

**Variable**	**Estimate**	**SE**	**OR**	**95 % CI**	**P-value**[a]
Sex (m)	-0.287	0.146	0.751	0.563 – 0.999	**0.049**
Age*	-0.542	0.114	0.784	0.722 - 0.851	**<0.001**
Immunocompromising diseases/drugs (y)	-0.071	0.389	0.931	0.434 – 1.997	0.855
LB in the past (y)	-0.262	0.175	0.769	0.546 – 1.083	0.133
Antibiotic prior to LBOC visit (y)	-0.662	1.165	0.517	0.053 – 5.068	0.571
Duration of EM till LBOC visit (days)	-0.003	0.0021	0.997	0.993 – 1.003	0.956
Diameter of EM (cm)	0.0003	0.005	1.00	0.991 – 1.006	0.135
EM type (multiple)	1.234	0.191	3.435	2.374 -5.030	**<0.001**
Isolation of *Borrelia* from skin (y)	-0.164	0.157	0.849	0.624 – 1.154	0.297

SE = standard error; OR = odds ratio; CI = confidence interval; EM = erythema migrans; LB = Lyme borreliosis; LBOC = Lyme borreliosis outpatient clinic; (y) = yes; (m) = male.

[a] Two-sided coefficient-level Wald p-values from the multivariable model; ORs are mutually adjusted for all other covariates in the same model. No adjustment for multiple comparisons was applied. Borderline and negative results were combined in the non-positive outcome category for binary analyses.

* estimation for 10 years (Age was modelled per 10-year increase. The odds ratio of 0.784 indicates that with each additional 10 years of age, the odds of the outcome decrease by approximately 22%).

Supplementary Table S5. Multivariable logistic regression analysis of factors associated with IgG seropositivity at the initial visit to Lyme Borreliosis Outpatient Clinic

**Variable**	**Estimate**	**SE**	**OR**	**95% CI**	**P-value**[a]
Sex (m)	0.929	0.145	1.144	0.861 – 1.522	0.353
Age*	0.119	0.040	1.127	1.042 – 1.219	**0.005**
Immunocompromising diseases/drugs (y)	-1.193	0.376	0.639	0.303 – 1.341	0.234
LB in the past (y)	2.342	0.175	1.508	1.072 – 2.134	**0.019**
Antibiotic prior to LBOC visit (y)	0.01	1.014	1.011	0.119 – 8.634	0.992
Duration of EM till LBOC visit (days)	3.049	0.003	1.009	1.003 – 1.015	**0.002**
Diameter of EM (cm)	9.295	0.007	1.073	1.05 – 1.077	**<0.001**
EM type (multiple)	5.368	0.21	3.096	2.071 – 4.737	**<0.001**
Isolation of *Borrelia* from skin (y)	-0.09	0.156	0.986	0.727 – 1.339	0.928

SE = standard error; OR = odds ratio; CI = confidence interval; EM = erythema migrans; LB = Lyme borreliosis; LBOC = Lyme borreliosis outpatient clinic; (y) = yes; (m) = male.

[a] Two-sided coefficient-level Wald p-values from the multivariable model; ORs are mutually adjusted for all other covariates in the same model. No adjustment for multiple comparisons was applied. Borderline and negative results were combined in the non-positive outcome category for binary analyses.

*estimation for 10 years (Age was modelled per 10-year increase. The odds ratio of 1. 127 indicates that with each additional 10 years of age, the odds of the outcome increase by approximately 13%).

Supplementary Table S6. Serologic findings in patients with erythema migrans approximately 2 months after the initial examination and the initiation of antibiotic treatment

	**EM** (*n* = 1356)	**Solitary EM** (*n* = 1111)	**Multiple EM** (*n* = 245)	**P – value**[a]
**Seropositivity**				
IgM	519 (38.3%)	365 (32.9 %)	154 (62.9 %)	**< 0.001**
IgG	724 (53.4%)	554 (49.9%)	170 (69.4%)	**< 0.001**
IgM and/or IgG	932 (68.7%)	721 (64.9%)	211 (86.1%)	**< 0.001**
**Numerical AU/mL values**				
IgM	14.5 (7.0 – 37.0)	12.8 (6.7 – 28.3)	39.9 (10.5 – 108.8)	**< 0.001**
IgG	18.2 (4.9 – 59.1)	14.9 (4.9 – 52.2)	37.2 (10.7 – 86.6)	**< 0.001**
IgM[b]	45.5 (31.2 – 104.5)	40.3 (28.4 – 75.2)	86.8 (43.1 – 190.0)	**< 0.001**
IgG[c]	54.0 (28.0 – 132.3)	52.2 (27.6 – 127.2)	60.0 (35.3 – 136.1)	**< 0.001**

Data are shown as number (%) or median (interquartile range).

EM, erythema migrans

[a] Comparison of findings in patients with solitary and multiple erythema migrans.

[b] Numerical AU/mL assay values among 519 IgM-positive patients, of whom 365 had solitary EM and 154 multiple EM. AU/mL values are assay-specific arbitrary units and should not be interpreted as absolute antibody concentrations.

[c] Numerical AU/mL assay values among 724 IgG-positive patients, of whom 554 had solitary EM and 170 multiple EM. AU/mL values are assay-specific arbitrary units and should not be interpreted as absolute antibody concentrations.

Supplementary Table S7. Multivariable logistic regression analysis of factors associated with IgM seropositivity 2 months later (i.e., 2 months after the onset of antibiotic treatment)

**Variable**	**Estimate**	**SE**	**OR**	**95 % CI**	**P-value**[a]
Sex (m)	-0.309	0.149	0.734	0.549 – 0.983	**0.038**
Age*	-0.210	0.040	0.811	0.752 –0.874	**<0.001**
Immunocompromising diseases/drugs (y)	0.338	0.388	1.402	0.656 – 2.996	0.383
LB in the past (y)	-0.246	0.179	0.782	0.551 – 1.109	0.168
Duration of EM till LBOC visit (days)	-0.003	0.003	0.997	0.992 – 1.002	0.290
Diameter of EM (cm)	-0.011	0.006	0.99	0.979 – 1.001	0.068
EM type (multiple)	1.240	0.196	3.457	2.355 – 5.073	**<0.001**
Isolation of *Borrelia* from skin (y)	-0.216	0.161	0.806	0.588 – 1.104	0.179
Antibiotic doxycycline (y)	-0.283	0.190	0.753	0.519 – 1.094	0.136
Duration of EM after antibiotic (days)	-0.006	0.004	0.994	0.987 – 1.000	0.065

SE = standard error; OR = odds ratio; CI = confidence interval; EM = erythema migrans; LB = Lyme borreliosis; (y) = yes; (m) = male.

[a] Two-sided coefficient-level Wald p-values from the multivariable model; ORs are mutually adjusted for all other covariates in the same model. No adjustment for multiple comparisons was applied. Borderline and negative results were combined in the non-positive outcome category for binary analyses.

*estimation for 10 years (Age was modelled per 10-year increase. The odds ratio of 0.811 indicates that with each additional 10 years of age, the odds of the outcome decrease by approximately 19%).

Supplementary Table S8. Multivariable logistic regression analysis of factors associated with IgG seropositivity 2 months later (i.e., 2 months after the onset of antibiotic treatment)

**Variable**	**Estimate**	**SE**	**OR**	**95 % CI**	**P-value**[a]
Sex (m)	0.166	0.145	1.180	0.888 – 1.569	0.254
Age*	0.110	0.040	1.116	1.032 – 1.206	**0.007**
Immunocompromising diseases/drugs (y)	-0.239	0.380	0.787	0.374 – 1.656	0.528
LB in the past (y)	0.763	0.179	2.145	1.511 – 3.046	**<0.001**
Duration of EM till LBOC visit (days)	0.008	0.003	1.008	1.002 – 1.013	**0.008**
Diameter of EM (cm)	0.046	0.006	1.047	1.034 – 1.060	**<0.001**
EM type (multiple)	1.000	0.207	2.719	1.811 – 4.082	**<0.001**
Isolation of *Borrelia* from skin (y)	0.123	0.156	1.131	0.833 – 1.537	0.430
Antibiotic doxycycline (y)	-0.088	0.186	0.915	0.636 – 1.319	0.635
Duration of EM after antibiotic (days)	0.007	0.003	1.007	1.000 – 1.013	**0.045**

SE = standard error; OR = odds ratio; CI = confidence interval; EM = erythema migrans; LB = Lyme borreliosis;; (y) = yes; (m) = male.

[a] Two-sided coefficient-level Wald p-values from the multivariable model; ORs are mutually adjusted for all other covariates in the same model. No adjustment for multiple comparisons was applied. Borderline and negative results were combined in the non-positive outcome category for binary analyses.

*estimation for 10 years (Age was modelled per 10-year increase. The odds ratio of 1.116 indicates that with each additional 10 years of age, the odds of the outcome increase by approximately 12%).

Supplementary Table S9. Paired comparison of seropositivity at baseline and at the follow-up visit approximately two months later

EM group	IgM baseline, n (%)	IgM follow-up, n (%)	IgM χ^2 (df=1); p-value	IgG baseline, n (%)	IgG follow-up, n (%)	IgG χ^2 (df=1); p-value
All (*n* = 1356)	563 (41.5)	519 (38.3)	10.52; 0.001	752 (55.5)	724 (53.4)	4.90; 0.027
Solitary EM (*n* = 1111)	397 (35.7)	365 (32.9)	6.17; 0.013	576 (51.8)	554 (49.9)	3.78; 0.052
Multiple EM (*n* = 245)	166 (67.8)	154 (62.9)	8.00; 0.005	176 (71.8)	170 (69.4)	1.13; 0.289

Data are n (%). Baseline and follow-up seropositivity were compared using McNemar's test for paired data without continuity correction; borderline and negative results were combined in the non-positive category for the primary binary analysis. In a sensitivity analysis restricted to patients with strictly positive or strictly negative results at both visits, excluding borderline results, neither overall change was statistically significant (IgM: chi-square = 2.21, df = 1, p = 0.138; IgG: chi-square = 0.30, df = 1, p = 0.583).

Supplementary statistical note. Post hoc diagnostics found no evidence of problematic multicollinearity (all VIF values <=1.291). Natural cubic-spline comparisons indicated evidence of nonlinearity for EM duration and lesion diameter in the baseline IgM and IgG models, for age in the follow-up IgM model, and for EM duration and lesion diameter in the follow-up IgG model. These diagnostics indicate that simple per-unit linear ORs should be interpreted as average linear summaries rather than as constant effects over the entire observed range.
